# The association between MAD2 and prognosis in cancer: a systematic review and meta-analyses

**DOI:** 10.18632/oncotarget.18414

**Published:** 2017-06-08

**Authors:** Tara Byrne, Helen G. Coleman, Janine A. Cooper, W. Glenn McCluggage, Amanda McCann, Fiona Furlong

**Affiliations:** ^1^ School of Pharmacy, Queen's University Belfast, Belfast, Northern Ireland, UK; ^2^ Centre for Public Health, Queen's University Belfast, Belfast, Northern Ireland, UK; ^3^ Department of Pathology, Belfast Health and Social Care Trust, Belfast, Northern Ireland, UK; ^4^ UCD School of Medicine, College of Health and Agricultural Science, University College Dublin, Dublin, Ireland, UK; ^5^ UCD Conway Institute of Biomolecular and Biomedical Research, University College Dublin, Dublin, Ireland, UK

**Keywords:** MAD2, cancer, prognosis and systematic review

## Abstract

This systematic review and meta-analyses investigates the expression of the cell checkpoint regulator, mitotic arrest deficiency protein 2 (MAD2) in cancerous tissue and examines whether an association exists between MAD2 levels and cancer survival and recurrence. Studies investigating MAD2 expression in cancer tissue utilising immunohistochemistry (IHC) were identified by systematic literature searches of Medline, Embase and Web of Science databases by October 2015. Random effects meta-analyses were performed to generate pooled hazard ratios (HRs) with 95% confidence intervals (CIs) of overall and progression-free survival according to MAD2 expression. Forty-three studies were included in the overall review. In 33 studies investigating MAD2 expression by IHC in cancer tissue, a wide range of expression positivity (11–100%) was reported. Higher MAD2 expression was not associated with an increased risk of all-cause mortality in a range of cancers (pooled HR 1.35, 95% CI 0.97–1.87; *P* = 0.077, *n* = 15). However, when ovarian cancer studies were removed, a significant pooled HR of 1.59 for risk of all-cause mortality in other cancer patients with higher expressing MAD2 tumours was evident (95% CI, 1.17–2.17; *P* = 0.003, *n* = 12). In contrast, higher MAD2 expression was associated with significant decreased risk of all-cause mortality in ovarian cancer patients (pooled HR = 0.50, 95% CI, 0.25–0.97; *P* = 0.04, *n* = 3). In conclusion, with the exception of ovarian cancer, increased MAD2 expression is associated with increased risk of all-cause mortality and recurrence in cancer. For ovarian cancer, reduced levels of MAD2 are associated with poorer outcome. Further studies are critical to assess the clinical utility of a MAD2 IHC biomarker.

## INTRODUCTION

The mitotic arrest deficiency protein 2 (MAD2) is an essential spindle assembly checkpoint (SAC) monitoring accurate chromosomal alignment at the metaphase plate before mitosis [1]. Much evidence implicates the overexpression of MAD2 as a driver of chromosomal instability in many cancer types [2–5]. Tumour formation resulting from loss of one of the MAD2 alleles suggests haploinsufficiency at this locus which is common to other SAC proteins [6]. MAD2 also functions beyond the SAC where it is known to interact with a number of checkpoint-unrelated proteins such as insulin receptor, estrogen receptor β and tumour necrosis factor α convertase [7–9]. Moreover, MAD2 influences cell proliferation, tumour metastasis and tumour recurrence [5].

MAD2 expression is regulated by a diverse range of cancer-associated proteins including the tumour suppressor protein p53, breast cancer 1 (BRCA1), the c-MYC oncogene, the checkpoint with forkhead and ring finger domains, E3 ubiquitin protein ligase (CHFR), E2 factor (E2F) and numerous microRNAs [10–16]. Moreover, MAD2 is a ubiquitously expressed protein, expressed by most tissues of the body, found localised in the nuclear, perinuclear and cytoplasmic compartments [17–19]. It is reliably measured by real-time PCR, western blots or immunohistochemistry (IHC) methods [19, 20]. The majority of studies predominantly evaluate MAD2 expression by IHC.

The association between MAD2 IHC expression and survival has been examined in a diverse range of carcinomas including colorectal, bladder, testicular, breast and ovarian, where both overexpression and low levels of MAD2 expression have been associated with survival, depending on the tumour type [20, 21]. However, inconsistencies in terms of study design, staining site analysed, expression cut-offs and study quality have precluded the interpretations of these study results, and the evaluation of MAD2 as a potential prognostic biomarker of patient survival. Therefore, there is an unmet need to systematically review published data relating to the extent of MAD2 expression in various tissues and the relationship between MAD2 expression evaluated through IHC and cancer prognosis.

The primary aim of this novel review and meta-analyses was to systematically identify research papers that analysed the association between MAD2 IHC staining and cancer patient survival and/or recurrence, and from these studies elucidate whether there is an association between MAD2 expression and cancer patient prognosis by conducting a meta-analysis.

## RESULTS

**Figure 1:**
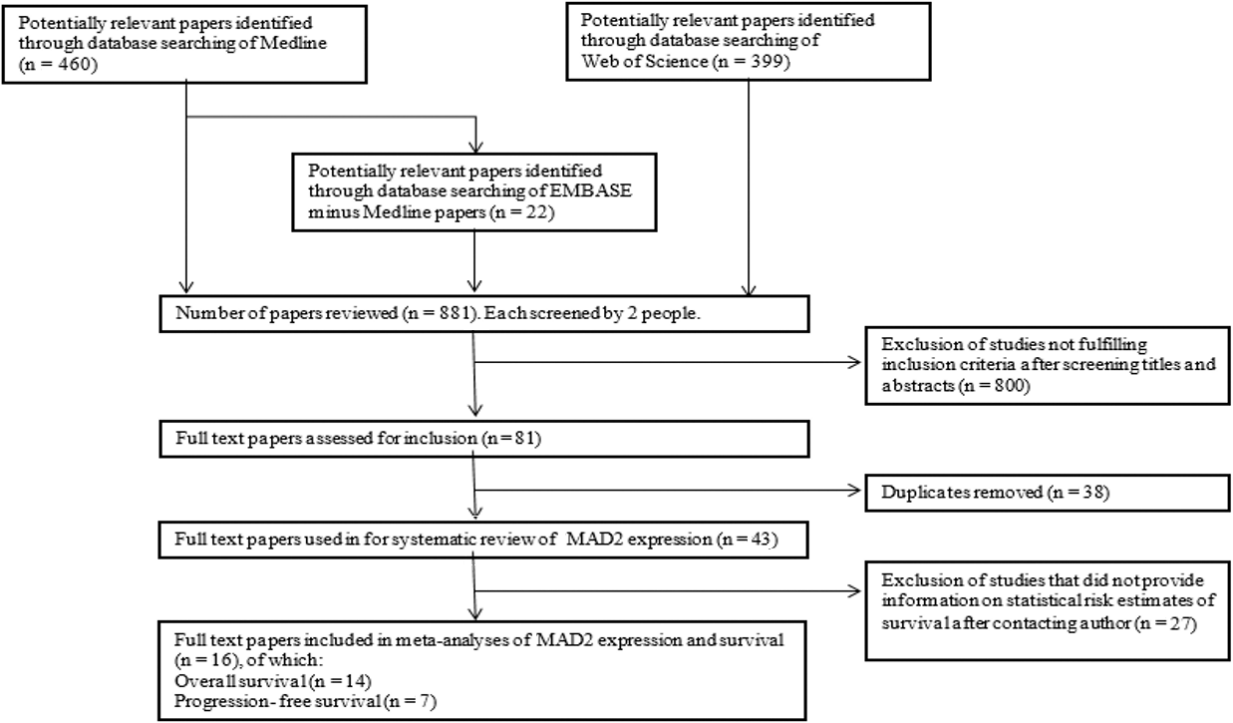
Flowchart of study selection that assessed MAD2 immunohistochemistry in cancer Three databases (Medline, Embase and Web of Science) were utilised to identify potential papers for inclusion in meta-analysis. After removal of duplicates and studies not fulfilling inclusion criteria, data was extracted from these full text papers to be included in meta-analyses evaluating MAD2 percentage expression and both progression-free and overall survival in relation to MAD2 expression.

The literature search identified 881 citations for review (Medline *n* = 460, Embase minus Medline *n* = 22 and Web of Science *n* = 399). As shown in Figure 1, after exclusion of publications following screening for title and abstract by reviewers, 81 papers remained and after removal of duplicates, 43 studies were fully screened and included in the review.

### MAD2 expression in cancerous tissue

A total of 33 studies provided expression analysis of MAD2 where expression of positivity ranged from 11.1 to 100% in cancerous tissue. Difficulties arose in the comparison of studies due to the fact that the majority of cancer types only have one study. In cancer types where more than one study has been undertaken, comparisons between studies were extremely complicated. This was as a result of the different expression methods utilised and the expression reported as different measures of central tendency (Supplementary Table 1).

### Association with overall survival

As shown in Table 1, studies included in this meta-analysis were published between 2008 and 2015, with nine studies originating from East Asia, three studies from Europe, one study from Brazil and one study from the USA. Cancer sample cohorts ranged from 27 to 400. The mean follow-up times, ranged from 3 to 3.29 years with median follow-up times ranging from 1.6 to 5.04 year. MAD2 IHC was mostly evaluated on tissue microarrays (TMAs), one study used both TMAs and full face sections, while six studies did not report the sample type assessed. Monoclonal antibodies were reported in eight studies, three studies reported the use of polyclonal antibodies and three studies did not report the type of antibody used. The cellular location investigated for MAD2 staining was nuclear in six studies, cytoplasmic for two studies, both nuclear and cytoplasmic in five studies and unreported in one study (Table 1). In the IHC staining analysis of MAD2 expression, four studies reported the involvement of a pathologist in the scoring of the IHC slides while 10 studies did not state if a pathologist assisted in the scoring. Two assessors determined MAD2 IHC staining in six studies, a single pathologist assessed MAD2 IHC scoring in one study and the remaining seven studies did not state the number of assessors.

**Table 1:** Characteristics of studies assessing the expression of MAD2 immunohistochemistry in cancer

Author	Year	Cancer site(s)	Samples assessed	Age range, years (mean or median)	Sex	Number of normal tissue samples	Number of cancer samples	Site of staining	Expression analysis	Survival analysis
Agosten	2007	Breast	TMA	NR	Females	NR	85	Perinuclear/cytoplasmic	✓	X
Burum-Auensen	2008	Colorectal	TMA	35–88 (68)	Males & females	17	55	Nuclear	✓	✓
Burum-Auensen	2007	Colorectal (UC-related)	TMA	NR	NR	10	8	Nuclear/additional staining was cytoplasmic	✓	X
Burum-Auensen	2010	Testicular	TMA	NR	Males	21	336	Nuclear with weak staining of cytoplasm	✓	X
Choi	2013	Bladder	TMA	23–97 (68)	Males & females	NR	339	Nuclear & cytoplasmic	✓	✓
Du	2011	Breast	NR	33–83 (54)	Females	NR	117	Nuclear/sometimes cytoplasmic	✓	X
Fung	2007	Testicular	NR	24–67 (37.7)	Males	12	23	Nuclear & cytoplasmic	✓	X
Furlong	2012	Ovarian	TMA and full face	NR	Females	NR	82	Nuclear	✓	✓
Genga	2015	Myelodysplastic syndrome	NR	18–91 (59.5)	Males & females	10	40	Cytoplasmic	✓	✓
Gladhaug	2010	Pancreatic	TMA	NR	Males & females	NR	218	Nuclear	✓	✓
Hannisdal	2010	Tonsillar	TMA	43 aged < 60 57 aged ≥ 60	Males & females	NR	105	Nuclear & cytoplasmic	✓	✓
Hisaoka	2008	Sarcoma	NR	NR	NR	50	50	Nuclear	✓	X
Kato	2012	Lung	TMA	123 aged < 60 239 aged ≥ 60	Males & females	NR	362	NR	✓	X
Kato	2011	Lung	TMA	26–87 (63.5)	Males & females	NR	358	Nucleoplasmic	✓	✓
Kim	2014	Uterine cervical	TMA	22–82 (44.1)	Females	100	232	Nuclear & cytoplasmic	✓	✓
Ko	2010	Salivary duct	NR	42–82 (58.5)	Males & females	NR	27	Cytoplasmic	✓	✓
Li	2013	Endometrial	NR	35–85 (51)	Females	30 normal, 30 hyperplasia	63	Nuclear & cytoplasmic	✓	✓
Li	2003	Colorectal	NR	28–81 (54.8)	Males & females	38	38	Brown-yellow staining mainly in cell plasma	✓	X
Li	2004	Colorectal	NR	25–79 (52.5)	Males & females	40	40	Brown-yellow staining mainly in cell plasma	✓	✓
Liao	2013	Cervical	NR	23–65 (43.5)	Females	10	90	Nuclear & cytoplasmic	✓	X
McGrogan	2014	Ovarian	TMA	32–77 (52)	Females	NA	72	Nuclear	✓	✓
Morishta	2012	Uterine cervical	NR	22–69	Females	NA	53	Nuclear	✓	X
Nakano	2012	Ovarian serous	NR	26–82	Females	NA	41	Nuclear	✓	✓
Nakano	2012	Ovarian mucinous	NR	13–88	Females	30	98	Nuclear	✓	✓
Park	2013	Ovarian	NR	59 aged < 60 26 aged ≥ 60	Females	NA	85	Nuclear 7 cytoplasmic	✓	✓
Rizzardi	2014	Oral SCC	NR	44–86 (61)	Males & females	NA	49	Nuclear	✓	✓
Sotillo	2006	Various	TMA	NR	NR	NA	351	Nuclear	✓	X
Suraokar	2014	Mesothelioma	TMA	NR	NR	NA	80	Nuclear & cytoplasmic	✓	✓
Teixeira	2015	Oral cancer	NR	25 aged < 62, 29 aged ≥ 62	Males & females	NA	54	Nuclear & cytoplasmic	✓	✓
Thoma	2009	Renal	TMA	NR	NR	NA	> 200	Nuclear	✓	X
Uemura	2009	Esophageal	TMA	32 aged < 60, 28 aged ≥ 60	Males & females	60	60	Nuclear & cytoplasmic	✓	X
Wang	2009	Gastric	TMA	NR	Males & females	102	102	Nuclear & cytoplasmic	✓	✓
Yu	2010	Osteosarcoma	NR	12–54 (25.2)	Males & females	20	48	Nuclear & cytoplasmic	✓	✓
Zhang	2008	Hepatocellular	TMA	184 <median age, 216> median age	Males & females	NR	400	Nuclear	X	✓
Zhao	2014	Endometrial	NR	NR (50.8)	Females	30	63	Nuclear & cytoplasmic	✓	X

NA = not applicable, NR = not reported, TMA = tissue microarray, UC = Ulcerative Colitis.

**Figure 2:**
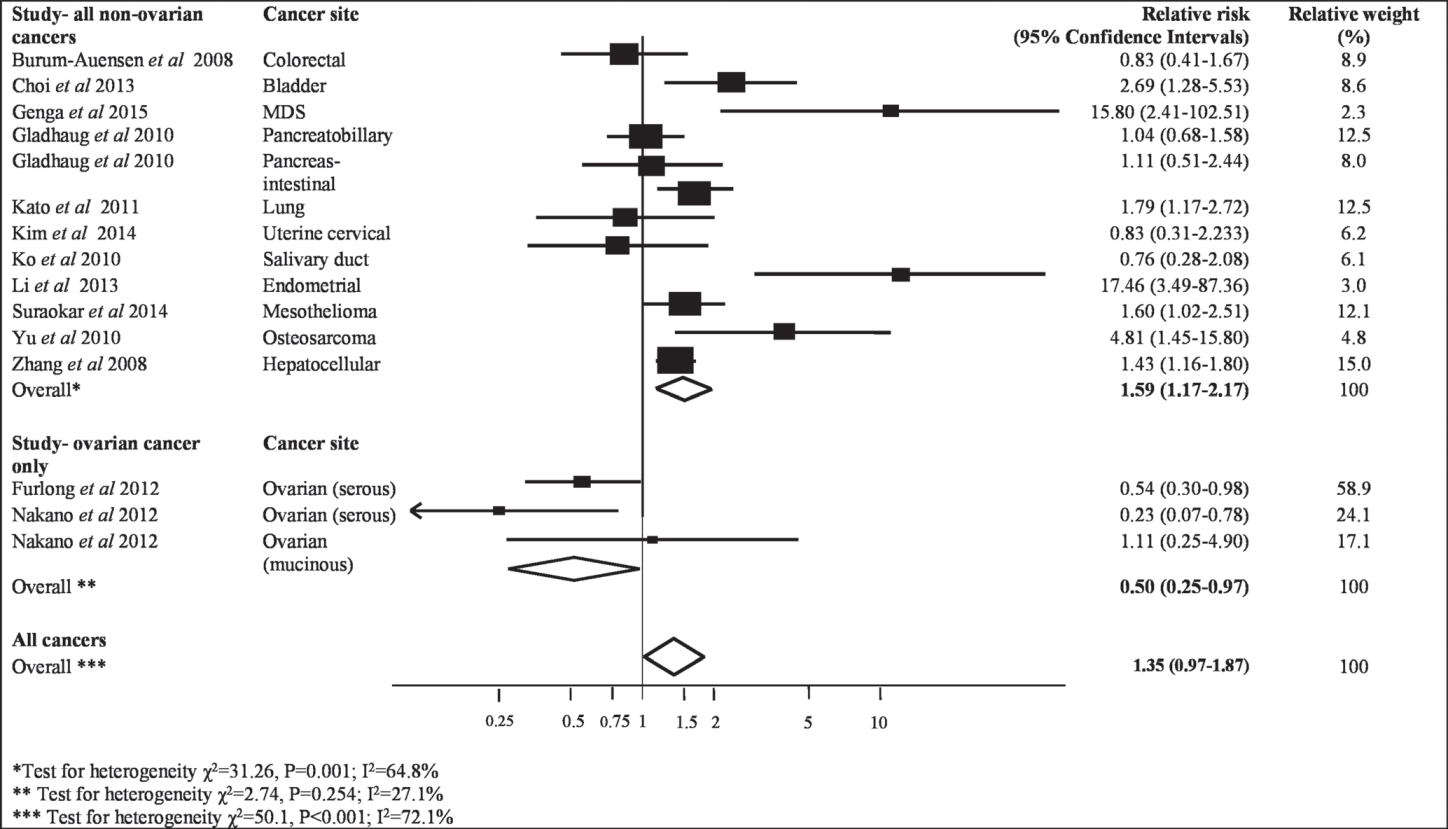
Meta-analysis of highest v. lowest category of MAD2 protein levels and risk to overall cancer survival

Sufficient data was provided by 14 studies, which contained 2318 patient samples to be included in the meta-analysis, to assess the association between MAD2 expression and overall survival (Table 2). As shown in Figure 2, higher MAD2 expression was associated with a non-significant increased risk of all-cause mortality in a range of cancers (pooled HR 1.35, 95% CI 0.97–1.87; *P* = 0.08). This investigation had an *I*^2^ value of 72% (*P <* 0.001), highlighting the substantial heterogeneity between studies, which was not markedly reduced in sensitivity analyses excluding individual studies (Figure 2).

**Table 2:** Descriptive characteristics and results of studies investigating MAD2 expression and cancer progression

Author	Cancer site(s)	Comparison (definition)^*^	Total number of patients/deaths	Mean (max.) follow-up, yrs	Relative risk (95% CI) Overall/cancer-specific survival	Relative risk (95% CI) Recurrence/disease free survival	Adjustments
Burum-Auensen	Colorectal	High v. low (≥ v. < median %)	55/NR	NR	HR 0.83, 95%CI 0.41–1.66, *P =* 0.59		Multivariate- variables NR
Choi	Bladder	High v. low (>3% v. ≤ 3%, determined by receiver operating characteristic curves)	339/29	Mean NR, median was given = 3.08 (15.25)	HR 2.68, 95% CI 1.29–5.55, *P =* 0.008		Unadjusted
Furlong	Ovarian	High v. low (intensity 3–4 v. 1–2)	82/NR	NR	HR 0.54, 95% CI 0.30–0.98, *P =* 0.041	HR 0.43, 95% CI 0.24–0.76, *P =* 0.004	Stage and grade
Genga	Myelodysplastic syndrome	High v. low (≥ 50% v. zero/< 50%)	40/10	3.24 ± 1.7 (5.75)	HR 15.79, 95% CI 2.42–102.86, *P =* 0.004		Age, gender, International Prognostic Scoring System at diagnosis
Gladhaug	Pancreatobiliary	High v. low (positive v. negative)	145/124	Mean NR, median was given = 1.6 (5)			Unadjusted
	Pancreas - Intestinal	High v. low (positive v. negative)	73/35	Mean NR, median was given = 1.6 (5)	HR 1.11, 95% CI 0.51–2.43, *P =* 0.786		Unadjusted
Kato	Lung	High v low (≥ 4 v. ≤ 3)	358/NR	Mean NR, median given = 5.04 (13.82)	HR 1.78, 95% CI 1.17–2.71, *P =* 0.008	HR 1.78, 95% CI 1.17–2.71, *P =* 0.008	Age, sex, pT status, pN status, pleural invasion, histological type, smoking history
Kim	Uterine cervical	High v. low (≥ 25% v. < 25%)	232/NR	Mean NR, median was given = 2.75 (10.25)		HR 0.83, 95% CI 0.31–2.23, *P =* 0.711	Unadjusted
Ko	Salivary duct	High v. low (intensity 3–4 v. 1–2)	27/14	3.11 (9.75)	HR 0.76, 95% CI 0.28–2.08 *P =* 0.59	HR 0.84, 95% CI 0.28–2.51, *P =* 0.75	Unadjusted
Li	Endometrial	High v. low (positive v. negative)	63/7	3 (5.75)	OR 17.4, 95% CI 3.5–87.62, *P =* 0.041		Unadjusted
McGrogan	Ovarian	High vs. low (intensity 3–4 v. 1–2)	71/44	NR		HR 0.51, 95% CI 0.23–1.10, *P =* 0.09	Tumour stage, grade, and optimal debulking
Nakano	Ovarian serous	High v. low (mean weighted scores 8–12 v. 0–6)	41/20	NR	HR 0.23, 95% CI 0.07–0.78, *P =* 0.019	HR 0.40, 95% CI 0.15–1.08, *P =* 0.069	Unadjusted
Nakano	Ovarian mucinous	High v. low (mean weighted scores 8–12 v. 2–6)	128/NR	NR	HR 1.10, 95% CI 0.25–4.90, *P =* 0.903		Unadjusted
Park	Ovarian	High v. low (score ≥ 2 v. ≤ 1)	85/NR	2 (6.58)		HR 0.26, 95% CI 0.06–1.09, *P =* 0.07	Age, stage, ovarian cancer type, p53 expression
Suraokar	Mesothelioma	High v. low (median, ≥ v. < 44)	77/75	NR	HR 1.60, 95% CI 1.02–2.51, *P =* 0.09		Unadjusted
Yu	Osteosarcoma	High v. low (strong v. weak)	48/NR	NR (9)	HR 4.79, 95% CI 1.45–15.80, *P <* 0.01		Unadjusted
Zhang	Hepatocellular	High v. low ( ≥10 v. < 10%)	400/302	3.29 (7)	HR 1.44, 95% CI 1.16-1.80, *P <* 0.001		Unadjusted

CI = Confidence intervals, HR = Hazard ratio, NR = not reported, OR: Odds ratio.

Interestingly, when all ovarian cancer studies are removed, as shown in Figure 2, a significant pooled HR of 1.59 for risk of all-cause mortality in patients with high expressing MAD2 tumours was evident compared to those with low expressing MAD2 tumours (95% CI, 1.17–2.17; *P* = 0.003), but still incurred high heterogeneity (I^2^ = 65%, *P* = 0.001). When only ovarian studies were investigated as a separate entity, higher MAD2 expression was associated with a significantly decreased risk of all-cause mortality compared to those with low expressing MAD2 tumours (pooled HR 0.50, 95% CI, 0.25–0.97; *P* = 0.04). This meta-analysis had an I^2^ value of 27% (*P* = 0.25) indicating that the results of the studies are reliably consistent with mild heterogeneity (Figure 2). There was no evidence of publication bias in the funnel plot assessing overall survival for studies (*P* = 0.82, Supplementary Figure 1). However from undertaking the meta-analysis it is evident that the different ovarian cancer subtypes may respond differently to MAD2 expression levels. Low MAD2 levels correlated with poorer survival as evident in Figure 2. In mucinous ovarian cancers, a different outcome was obtained where low MAD2 levels correlate with decreased risk of all-cause death. However, the association between MAD2 levels and survival in mucinous cancers may not be valid as the HR is extremely close to 1 (HR = 1.11, 95% CI; 0.25–4.90) arising from a single study.

### Association with progression-free survival

The meta-analyses of progression-free survival included studies published between 2010 and 2014 with five studies from East Asia and two studies from Europe (Table 1). Cancer sample cohorts ranged from 27 to 358. The mean/median follow-up times reported ranged from 2–5 years.

The use of TMAs or full face sections for MAD2 IHC analysis was unreported in three studies. However, most other studies utilised TMAs with one study using both TMAs and full face sections. Monoclonal antibodies were reported in five studies with two studies not reporting the type used. MAD2 nuclear staining was reported in three studies, MAD2 cytoplasmic staining was reported in one study and both MAD2 nuclear and cytoplasmic staining assessed in two studies (Table 1). MAD2 IHC staining analysis was assessed by a pathologist in two studies, while five studies did not state if a pathologist assisted in the scoring. MAD2 IHC staining was determined by at least two assessors in four studies and three studies did not state the number of assessors.

**Figure 3:**
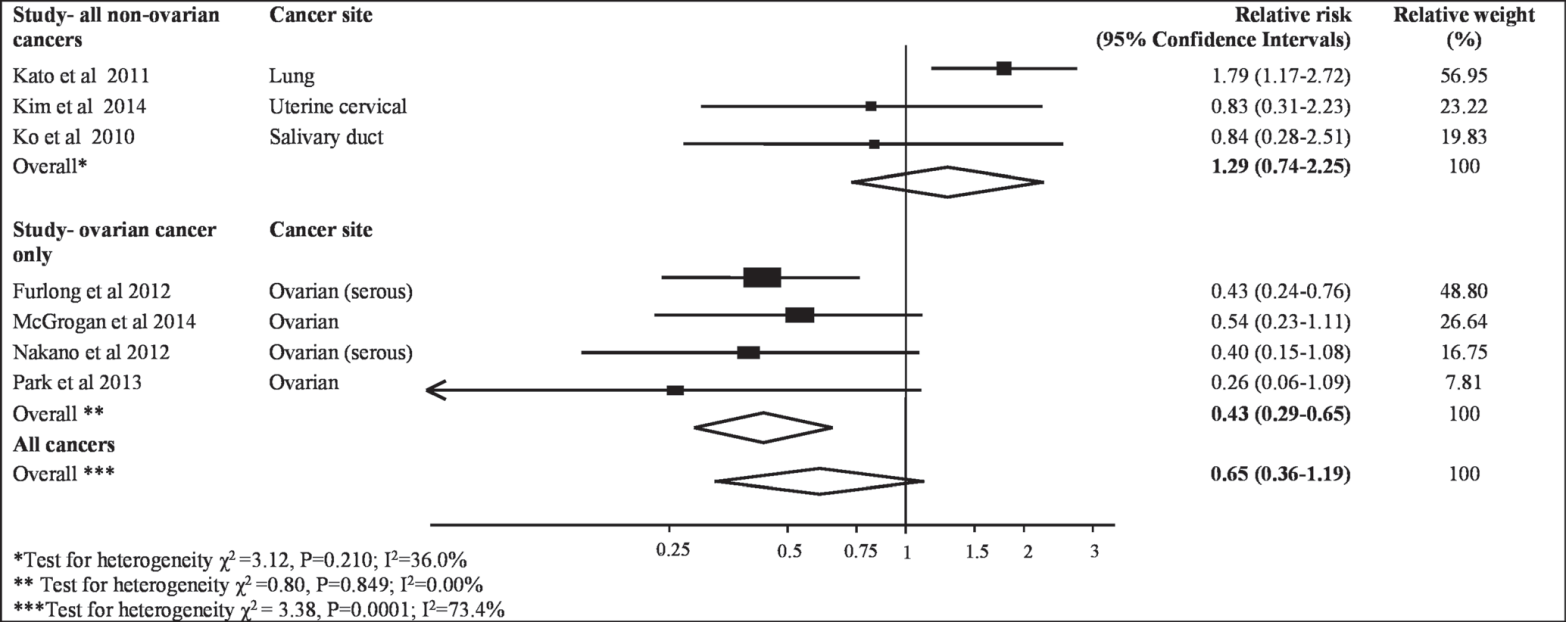
Meta-analysis of highest v. lowest category of MAD2 protein levels and risk to progression free cancer survival

The meta-analysis of the association between MAD2 expression and progression-free survival was assessed from seven studies containing 997 patient samples (Table 2). The results from all studies indicated that lower MAD2 expression was not significantly associated with an increased risk of cancer recurrence (pooled HR = 0.65, 95% CI, 0.36–1.19; *P* = 0.16, Figure 3). Similar to the association with overall survival, MAD2 expression is different in ovarian cancer compared to other cancers. A pooled HR for progression-free survival in patients with high-expressing non-ovarian MAD2 tumours compared to those with low expressing MAD2 tumours was 1.29 (95% CI, 0.74–2.25; *P* = 0.38). Mild heterogeneity was observed (*I*^2^ value = 36%, *P* = 0.21). In contrast, a significant pooled HR of 0.43 (95% CI,0.29–0.65; *P <* 0.001) was evident for progression-free survival in patients with high expressing MAD2 ovarian tumours compared to those with low expressing MAD2 tumours. An I^2^ value of 0% was seen thereby identifying the results as consistent (*P* = 0.85). Moreover, the funnel plots for these analyses had minimal asymmetry, indicating no publication bias (*P* = 0.09, Supplementary Figure 1).

## DISCUSSION

The aim of this novel systematic review with meta-analyses was to summarise the extent of MAD2 expression in various tissues, and to investigate the association between MAD2 IHC expression and both overall and progression-free survival in a diverse range of cancers. The results highlight that higher MAD2 expression is associated with poorer survival in cancer patients, with the exception of ovarian cancer, where conversely, higher MAD2 expression is associated with improved survival.

Our results indicated that MAD2 dysregulation is heavily tissue dependent in cancerous tissue with MAD2 IHC levels ranging from 11.1% to 100% in cancerous tissue. Dysregulation of MAD2 levels by either up- or down-regulation of the gene can result in similar genomic aberrations and contribute to poorer patient survival [14, 40]. From our results, it is evident that high MAD2 levels are associated with increased risk of all-cause death and cancer recurrence in non-ovarian cancers while low MAD2 levels are associate with poorer patient survival in ovarian cancer. Through *in vitro* and *in vivo* studies, high MAD2 levels are known to correlate with increased cellular proliferation, cellular transformation, migration, invasiveness and cancer metastasis which could contribute to this poorer patient outcome [41–44]. In addition, a number of studies have shown that low MAD2 levels correlate with resistance to commonly utilised anticancer agents such as taxol, DNA-damaging agents, vincristine and γ- irradiation. Therefore, it is not surprising that both low or high MAD2 levels can lead to a worsen patient prognosis [20, 45–48]. We previously reported that low MAD2 levels correlated with shorter progression-free survival in high grade serous ovarian cancer patients treated with platinum alone or in combination with paclitaxel and concluded this occurred as a result of increased resistance to paclitaxel [20].

In ovarian cancer, the association of high MAD2 expression and survival is in contrast to the reported association of low MAD2 expression in the other cancer types included in this study and raises many questions about why MAD2 levels in ovarian cancer produce such a different response to patient survival. Currently, there is no explanation for this outcome. However, one possible explanation could be linked to the fact that unlike most cancer types that metastasise mainly via haematogenous or lymphatic routes, ovarian cancer, especially the most common and lethal type high grade serous carcinoma, metastasises through peritoneal dissemination [49]. Therefore as ovarian cancers progress and metastasise, they may possibly become less reliant on MAD2 expression as previously reported in osteosarcoma [33]. Another possibility for the dysregulation of MAD2 expression could be related to the regulation of MAD2 by BRCA1 [11]. BRCA1, which is mutated in 10% of ovarian cancers, is a transcriptional regulator of MAD2 and the aberrant activity of mutant BRCA1 in ovarian cancer may lead to altered transcriptional regulation of MAD2 expression [11, 50]. Furthermore, ovarian tumours with high MAD2 levels were previously shown to be associated with increased sensitivity to taxol and improved patient survival, which may indicate that taxol response in ovarian tumours is possibly more dependent on the SAC activity of MAD2 than other cancer types [20, 51].

From the meta-analysis, the different subtypes of ovarian cancer have distinctive survival responses in terms of MAD2 expression. Studies of high grade serous ovarian cancer have shown that low MAD2 levels lead to poorer patient survival. In contrast, mucinous ovarian cancer appears to have a weaker inverse association between MAD2 levels and survival. A possible reasoning for this is that p53 and BRCA1, known regulators of MAD2, are commonly mutated in high serous ovarian cancer (*Tp53* mutation is ubiquitous in ovarian high grade serous carcinoma) and mutations in these genes are uncommon in other ovarian cancer subtypes such as mucinous, clear cell, endometrioid and low grade serous [11, 52]. Similarly in breast cancer, where p53 and BRCA1 are also regularly mutated, high MAD2 levels are associated with improved breast cancer specific survival [53]. Additionally, high grade serous ovarian cancers express lower levels of E-cadherin and higher levels of N-cadherin compared to mucinous which is suggestive of a more epithelial-mesenchymal transition phenotype which in-turn triggers tumour metastasis and possibly reduce the requirement of MAD2 for tumour spread [54, 55]. However, due to the limited number of studies on MAD2 expression in ovarian cancer, we cannot conclude if different ovarian subtypes have different survival rates depending on MAD2 expression. Further analysis of MAD2 IHC expression in ovarian cancer may result in an IHC biomarker capable of stratifying the various ovarian cancers subtypes.

The heterogeneity in the meta-analysis of the correlation of MAD2 expression and overall survival in non-ovarian cancer studies was considerable between studies (64.8%). This could be attributable to the methodological diversity between studies, including sub-cellular location evaluated, scoring method utilised, expression threshold chosen and cancer site investigated. However, heterogeneity associated with meta-analyses of MAD2 expression and ovarian cancer prognosis was low.

The primary strength of this review lies in its ability to determine the effect of MAD2 expression on cancer survival for the first time, taken from a robust search of three databases for relevant studies. We were also able to standardise comparator groups, displaying all results as high v. low MAD2 levels, which overcomes potential confusion when comparing results from studies reporting high v. low or low v. high comparisons of MAD2 expression in relation to progression. The utility of prognostic markers are relevant to both understanding the mechanisms of progression and the ability to predict patient outcome (who may benefit from more/less intensive treatments or follow-up regimens). MAD2 could also be combined with other cell cycle proteins such as P27, CDC20, BUBR1. However, there is limited evidence to suggest it would improve the prognostic ability of MAD2 [21, 32, 41, 56, 57]. Furthermore, there was no evidence of publication bias associated with the meta-analyses reported in this study which increases the robustness of the results.

One of the limitations of this review is that currently there is no standardised method for MAD2 IHC evaluation. Standardisation is vital across the entire IHC process from pre- to post-analytical stages and across laboratories to allow comparison between studies [58, 59]. This was particularly noticeable in the evaluation scoring system, where in some cases only MAD2 expression positivity was determined with the degree or percentage of MAD2 staining intensity not considered. Furthermore, the arbitrary cut-off chosen to indicate high MAD2 expression differs from study to study. Between-study variances may also have resulted from diverse populations utilised in these studies especially since a number of the studies were quite small. Standardisation of these factors across studies will allow more comparable studies and provide more robust results. Another limitation is that a meta-analysis for cancer-specific survival according to MAD2 expression was unable to be undertaken due to a lack of studies reporting on this outcome. Also the majority of the studies utilised in this review were unadjusted for confounders known to affect survival of cancer patients such as age and stage [60, 61]. It is therefore difficult to determine how age and stage would affect the association of MAD2 expression on patient survival [62–64]. Of note, in the study that adjusted for age and stage the authors demonstrated that the association of MAD2 expression and survival was independent of these two confounders [25]. Therefore, future studies to analyse MAD2 IHC expression and survival should adjust for known cofounders to improve the reliability of results. Furthermore, as the studies examining MAD2 expression by IHC have utilised different sample numbers, power analysis should be considered before the study is undertaken to ensure that the data correctly identifies a statistical significant difference [65, 66]. Tissue microarrays may make a greater number of samples more achievable [67]. Despite these limitations, the overall outcomes remained consistent.

Recommendations for future studies evaluating the association of MAD2 IHC expression include standardising the reporting of results investigating high vs. low MAD2 expression (the cut-offs may differ between different tumour types), and include required statistical values in order to undertake a meta-analysis such as HR and 95% CI. Additional studies should adjust for known clinical prognostic factors such as age and stage and also carry out power analysis. In this review, apart from ovarian cancer, there has only been one study in each cancer type that has met the required statistical findings in order to be included in a meta-analysis (Table 2). Thus, more studies in each cancer type are required to definitively determine if the correlation of MAD2 IHC expression with patient survival is cancer-specific. Furthermore, investigation of the molecular pathways involving MAD2 is essential to help researchers understand the mechanism behind the results of this review. Recent studies have also highlighted a potential therapeutic role for MAD2 where silencing of the MAD2 gene using siRNA lead to increased apoptotic cell death in a non-small cell lung cancer model [68]. Additionally, MAD2's role as a predictive biomarker could also be particularly important. Further studies of cancer samples from patients treated with and without taxol/DNA- damaging agents would evaluate if MAD2 expression could predict the efficacy of these treatments on patient survival.

In conclusion, this review illustrates the potential for MAD2 IHC as a prognostic biomarker in cancer, especially in ovarian cancer where there is approximately a 50% lower risk of death or tumour recurrence in patients in the higher MAD2 expression category compared to the lower. An opposing prognostic role for increased risk of death in other cancer sites displaying high MAD2 expression may exist. Further studies are critical to assess the clinical utility of a MAD2 IHC biomarker and the molecular pathways affected by MAD2 that may improve patient outcomes. We outline recommendations for the standardised conduct and reporting of MAD2 expression studies in relation to cancer progression outcomes.

## MATERIALS AND METHODS

This systematic review and meta-analysis adhered to the guidelines provided by the Preferred Reporting Items for Systematic Reviews and Meta-Analyses (PRISMA) statement [22].

### Search strategy

Systematic literature searches were performed using three electronic databases; Medline (US National Library of Medicine, National Institutes of Health, Bethesda, Maryland, USA), Embase (Reed Elsevier PLC, Amsterdam, The Netherlands) and Web of Science (Thomson Reuters, New York, USA) from their origin until week five October 2015. A systematic search to identify publications, in any language, was applied using the following keywords and/or Medical Subject Heading (MeSH) terms; (Mitotic Arrest Deficient-Like 1 OR MAD2 or HSMAD2 or MAD2-Like Protein 1 OR Mitotic Arrest Deficient 2-Like Protein 1 OR Mitotic Spindle Assembly Checkpoint Protein MAD2A) AND (carcinoma(s) OR cancer(s) OR neoplasm(s) OR tumo (u) r(s) OR adenocarcinoma(s)).

### Study selection

Inclusion criteria for studies were defined using the PICO model (population, intervention, control/comparison and outcome). The population included patients with any type of cancer. In order to be included in this systematic review, these studies needed to include the intervention which was an IHC method for the assessment of MAD2 expression. Studies must also have reported a comparison between high/positive and low/negative MAD2 levels in cancerous tissue. The outcome of the study must have reported either the proportion of tissue samples exhibiting MAD2 expression (or data from which this proportion could be calculated) and/or the statistical association between MAD2 and prognosis in cancer patients. Conference abstracts and poster presentations were excluded. Only human studies and literature available in the English language were included. Where publications reported on a sample source that was entirely overlapping and reported on the same outcomes, only the largest/most recent set of results was retained.

The principal reviewer (T. Byrne) screened all titles and abstracts, and two other reviewers (F. Furlong and J. Cooper) independently screened half of the titles and abstracts each. In cases where an abstract was unavailable or the article's significance was unclear, the full article was acquired for further examination. Those identified by any of the two reviewers for possible inclusion were brought forward for full text review.

### Data extraction

From the full text of identified articles, where available, the following data was extracted by the principal reviewer (T.Byrne): authors, year of publication, journal name, location and number of centres samples were obtained from, primary aim of the study, source and method which samples were obtained, number of participants, mean age and sex of participants, immunohistochemical methods, type of antibody, manufacturer and dilution of antibody, type of staining, scoring system, IHC assessment procedure, number of patients with positive/negative/weak/moderate/strong MAD2 expression tumours, follow-up time, survival, and/or recurrence results including reported hazard ratios (HRs), confidence intervals (CIs) and associated *p*-values. Authors from publications that did not have all required information were contacted to obtain information and summary-level data was utilised for meta-analysis.

### Statistical analysis

Meta-analyses were conducted to determine the association between MAD2 expression and overall survival and progression-free survival, where possible. Several of these studies did not present the results required for meta-analysis as high v. low comparisons of MAD2 expression. In the first instance authors were contacted to request information (provided by Furlong *et al*, Genga *et al*, Ko *et al*) [20, 23, 24]. For one study, Park *et al*, the HR for low v. high comparison was inverted in order to achieve comparability with studies where the HR for high v. low comparison was provided [25]. For other studies, the HR and 95% CI were derived by applying Parmar's methods [26] when the number of observed deaths in the high and low comparison groups had been presented together with the log-rank *p*-value for the associated Kaplan Meier curve (conducted for Li *et al*, Zhang *et al*, Nakano *et al* – serous and mucinous ovarian cancer studies, Suraokar *et al*, Kim, Choi, Yu, Gladhaug *et al* and Genga *et al* studies) [21, 23, 27–34]. Adjusted/unadjusted HRs and 95% CI (with the maximally adjusted rates used where possible) were combined and weighted to provide pooled estimates. A random effects model was utilised in order to account for the anticipated heterogeneity between studies. Subgroup analysis was possible by cancer type for ovarian cancer studies, where at least three studies had been published, but not for other cancer types. The extent of heterogeneity in the pooled estimates was calculated using the χ^2^ test and I*^2^* statistic. The I*^2^* statistic estimates the degree of variation between findings that is a result of heterogeneity rather than chance [35]. An I*^2^* statistic above 25%, 50% or 75% was decided to denote mild, moderate and high heterogeneity respectively [36]. In order to evaluate publication bias, funnel plots were constructed according to the Begg and Egger methods, by plotting relative risks to their corresponding standard errors [37, 38]. In addition, sensitivity analysis was conducted to determine excessive influences of studies by removing each study individually and assessing the effect on pooled estimates and the heterogeneity I*^2^* statistic [39]. Statistical analysis was conducted utilising Stata version 11.2 software (Stata Corporation, College Station, TX, USA).

## SUPPLEMENTARY MATERIALS FIGURE AND TABLES



## Abbreviations

MAD2: mitotic arrest deficiency protein 2
SAC: spindle assembly checkpoint
BRCA1: breast cancer 1
CHFR: Checkpoint with Forkhead And Ring Finger Domains, E3 Ubiquitin Protein Ligase
CIs: confidence intervals
E2F: E2 factor
HRs: hazard ratios
IHC: immunohistochemistry
MeSH: Medical Subject Heading
PRISMA: Preferred Reporting Items for Systematic Reviews and Meta-Analyses
TMAs: tissue microarrays
